# United Twins

**Published:** 1915-06

**Authors:** 


**United Twins.** The Houston Chronicle describes a local case of girls of Spanish descent, united by "a band of flesh 13 inches in circumference" taken to the Mayos for operation.

